# A Unique Protein Phosphatase with Kelch-Like Domains (PPKL) in *Plasmodium* Modulates Ookinete Differentiation, Motility and Invasion

**DOI:** 10.1371/journal.ppat.1002948

**Published:** 2012-09-20

**Authors:** David S. Guttery, Benoit Poulin, David J. P. Ferguson, Balázs Szöőr, Bill Wickstead, Paula L. Carroll, Chandra Ramakrishnan, Declan Brady, Eva-Maria Patzewitz, Ursula Straschil, Lev Solyakov, Judith L. Green, Robert E. Sinden, Andrew B. Tobin, Anthony A. Holder, Rita Tewari

**Affiliations:** 1 Centre for Genetics and Genomics, School of Biology, Queens Medical Centre, University of Nottingham, Nottingham, United Kingdom; 2 Nuffield Department of Clinical Laboratory Science, University of Oxford, John Radcliffe Hospital, Oxford, United Kingdom; 3 Centre for Immunity, Infection and Evolution, Institute of Immunology and Infection Research, School of Biological Sciences, University of Edinburgh, Edinburgh, United Kingdom; 4 Division of Cell and Molecular Biology, Imperial College London, London, United Kingdom; 5 Medical Research Council Toxicology Unit, Leicester, United Kingdom; 6 Division of Parasitology, MRC National Institute for Medical Research, Mill Hill, London, United Kingdom; Duke University, United States of America

## Abstract

Protein phosphorylation and dephosphorylation (catalysed by kinases and phosphatases, respectively) are post-translational modifications that play key roles in many eukaryotic signalling pathways, and are often deregulated in a number of pathological conditions in humans. In the malaria parasite *Plasmodium*, functional insights into its kinome have only recently been achieved, with over half being essential for blood stage development and another 14 kinases being essential for sexual development and mosquito transmission. However, functions for any of the plasmodial protein phosphatases are unknown. Here, we use reverse genetics in the rodent malaria model, *Plasmodium berghei*, to examine the role of a unique protein phosphatase containing kelch-like domains (termed PPKL) from a family related to *Arabidopsis* BSU1. Phylogenetic analysis confirmed that the family of BSU1-like proteins including PPKL is encoded in the genomes of land plants, green algae and alveolates, but not in other eukaryotic lineages. Furthermore, PPKL was observed in a distinct family, separate to the most closely-related phosphatase family, PP1. In our genetic approach, C-terminal GFP fusion with PPKL showed an active protein phosphatase preferentially expressed in female gametocytes and ookinetes. Deletion of the endogenous *ppkl* gene caused abnormal ookinete development and differentiation, and dissociated apical microtubules from the inner-membrane complex, generating an immotile phenotype and failure to invade the mosquito mid-gut epithelium. These observations were substantiated by changes in localisation of cytoskeletal tubulin and actin, and the micronemal protein CTRP in the knockout mutant as assessed by indirect immunofluorescence. Finally, increased mRNA expression of *dozi*, a RNA helicase vital to zygote development was observed in *ppkl^−^* mutants, with global phosphorylation studies of ookinete differentiation from 1.5–24 h post-fertilisation indicating major changes in the first hours of zygote development. Our work demonstrates a stage-specific essentiality of the unique PPKL enzyme, which modulates parasite differentiation, motility and transmission.

## Author Summary

Malaria parasites are single-celled organisms, which alternate their life-cycle between vertebrate and mosquito hosts. In the mosquito, the malaria parasite undergoes sexual development, whereby a male and female gamete fuse to form a zygote. This zygote then elongates into an invasive stage, termed an ookinete, which can glide to and penetrate the mosquito's gut wall in order to form a cyst (called an oocyst). Protein phosphorylation is known to play a vital role during this process; however, the role of *Plasmodium* kinases (which phosphorylate proteins) during zygote/ookinete maturation is better understood than the completely uncharacterised plasmodial phosphatases (which dephosphorylate proteins). Using a malaria parasite which infects mice, *Plasmodium berghei*, we show that a unique protein phosphatase containing kelch-like domains (called PPKL) plays a vital role in ookinete maturation and motility. Deleting this gene produces ookinetes whose shape is grossly abnormal, resulting in non-motile parasites that cannot penetrate the lining of the mosquito gut wall. Overall, PPKL is an essential phosphatase that is critical to ookinete development, motility and invasion.

## Introduction

Reversible protein phosphorylation is a ubiquitous regulatory process for a variety of eukaryotic and prokaryotic pathways, including cell-cycle regulation, cell to cell signalling, cell proliferation and differentiation [1]. In humans, aberrant regulation of protein phosphorylation has been implicated in cancers [2], and plays a central role in many other pathological diseases [3]. Protein phosphorylation and dephosphorylation is catalysed by protein kinases and phosphatases, respectively [4]–[7], and nearly two-thirds of the proteins encoded in the human genome are believed to be modified by reversible phosphorylation [8] at over 650,000 phosphosites (PhosphoNET KnowledgeBase (www.phosphonet.ca)), emphasizing the importance of this post-translational modification.

This regulatory mechanism is highly conserved [3], and plays a vital role in development of apicomplexan protozoan parasites of the genus *Plasmodium* [9], [10], which are globally responsible for over a million deaths annually through malaria [11]. The *Plasmodium* life-cycle proceeds via a number of distinct developmental stages: asexual exo-erythrocytic proliferation in liver hepatocytes and intra-erythrocytic multiplication in erythrocytes of the vertebrate host, and sexual development in the female *Anopheles* mosquito [12]. During both asexual and sexual development the parasite utilises a number of signalling pathways, many of which involve reversible protein phosphorylation. Systematic functional analyses of plasmodial protein kinases (PKs) in both the human parasite *P. falciparum* and the rodent model *P. berghei* species have revealed that over half of their kinome is essential to asexual blood stage schizogony [9], [10]. Furthermore, reverse genetic studies in *P. berghei* have shown that a further 14 PKs have specific functions during sexual development of the parasite within the mosquito mid-gut lumen and subsequent migration to the salivary glands [10], [13]–[21]. In particular, development of the motile and invasive ookinete within the mosquito mid-gut is known to be highly dependent upon two NIMA-related kinases, NEK2 and NEK4 [14], [15]. Of the three invasive stages of malaria parasite development (namely the sporozoite, merozoite and ookinete), the ookinete stage is unique in that it can develop extracellularly and lacks rhoptries, an organelle specifically associated with cell invasion. This is in contrast to the other “zoite” forms (merozoites and sporozoites) that develop intracellularly and contain both rhoptries and micronemes as apical organelles. However, all of these invasive stages comprise a unique cortical structure termed a pellicle, which consists of a parasitic plasma membrane and an underlying double membrane structure termed the inner membrane complex (IMC) [22]. Parasite motility is powered by an actin myosin motor termed the glideosome [23], which resides within the pellicle of invasive stages. At the molecular level, the motility and mid-gut invasion of the ookinete involves the secretion of a number of membrane proteins including the motor complex-associated proteins glideosome-associated protein 45 (GAP45) and myosin-A tail domain interacting protein (MTIP) [24], micronemal proteins such as circumsporozoite-and trap-related protein (CTRP) [25] and secreted ookinete adhesive protein (SOAP) [26], as well as calcium-dependent protein kinase 3 (CDPK3) [21].

Although the role of plasmodial kinases has been intensively studied, the role of all the complementary protein phosphatases (PPs) during any stage of *Plasmodium* development is unknown. Biochemical studies have shown that *P. falciparum* possesses predominantly phosphatase-1-like activities, and chemical inhibition of phosphatase activity by use of calyculin A and okadaic acid significantly reduces asexual blood stage proliferation [27]. In contrast to the human phosphatome (comprising approximately 156 phosphatases [28] (and PhosphoNET KnowledgeBase (www.phosphonet.ca)), the *Plasmodium* genome codes for one of the smallest phosphatomes of all the eukaryotic phyla known to date, with 27 putative protein phosphatases falling into four major classes: phosphoprotein phosphatases (PPPs), metallo-dependent protein phosphatases (PPMs), protein tyrosine phosphatases (PTPs) and NLI interacting factor-like phosphatases (NIFs) [28].

Like the kinases (although fewer in number), the *Plasmodium* phosphatome contains members that have no orthologues in mammalian systems [28]. The function of the *Shewanella*-like PPs (Shelphs) and the likely inactive pseudo-phosphatase EF-hand containing phosphatase (EFPP) is completely unknown [29]; however, in *P. falciparum*, Shelphs are postulated to have a role in erythrocyte invasion [30]. Furthermore, a distinctive PP1-related enzyme belonging to the PPP family of phosphatases comprising an N-terminal kelch repeat domain and a C-terminal PP1-like phosphatase domain (named PPKL: Protein Phosphatase with Kelch-Like domains) has been detected in the apicomplexans *Cryptosporidium hominis, Toxoplasma gondii* and *Theileria parva* (one gene per genome), as well as in the land plants *Arabidopsis thaliana* and *Oryza sativa* (4 and 5 genes respectively) [31], [32]. The kelch motif is widespread and involved in many cellular functions, particularly in actin-based cytoskeleton formation and transcriptional regulation [33]. PPKL itself has only been studied in detail in *Arabidopsis thaliana* (where it is known as BSU1 or *bri1* suppressor1) and along with the kinase BIN2, is involved in brassinosteroid hormone signalling and phosphorylation of the transcription factors BZR1 and BES1 [34]. Even though PPKL was first discovered in *P. falciparum* (initially named PfPPα), where RT-PCR analysis showed mRNA expression exclusively in gametocytes and hence suggesting a role during sexual development [35], its function has remained unknown. Transcriptomic and proteomic studies of *P. falciparum* [36] and *P. berghei* [37] showed high levels of *ppkl* transcripts in gametocytes and PPKL protein in ookinetes, respectively (PlasmoDB), although discordant results were found in a study of the proteome of sex-specific gametocytes [18].

In this study, we have used *P. berghei* to elucidate the function of the PPKL enzyme during the *Plasmodium* life-cycle. We show by using reverse genetics that *Plasmodium* PPKL has an essential function during ookinete differentiation, affecting apical end integrity, collar and pellicle morphology, and microtubule linkage to the IMC that are crucial for parasite shape, motility and invasion.

## Results

### PPKL is a unique Ser/Thr phosphatase with N-terminal kelch domains and is limited to *Alveolata* and *Viridiplantae*

**Figure 1:**
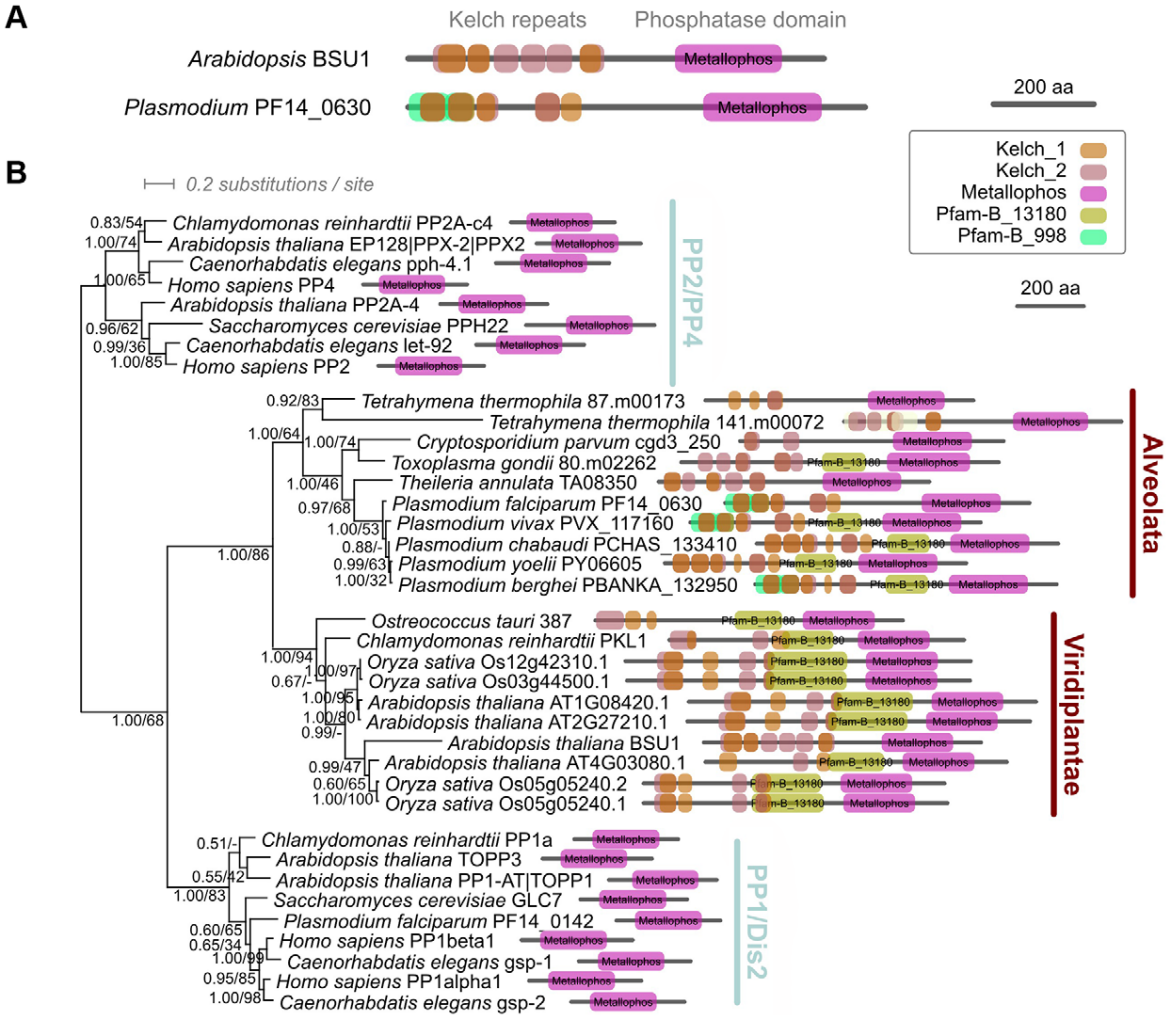
Phylogenetic analysis of *Plasmodium* PPKL. A. Schematic representations of the predicted protein architectures (Pfam domains) of the PPKL orthologues in *A. thaliana* and *P. falciparum*. B. Bayesian phylogeny of kelch-like phosphatases. Tree shown is the consensus of trees from four independent inferences based on a trimmed alignment of the phosphatase domain. Numbers beside nodes represent support from Bayesian posterior probabilities.

To identify PPKL-like phosphatases in *Plasmodium*, we used BSU1 from *Arabidopsis thaliana* to seed an iterative profile-based similarity search [38]. Phylogenetic analysis based on a common phosphoesterase domain (Pfam: PF00149) revealed a robust family of BSU1-like proteins encoded in the genomes of land plants, green algae and alveolates (Figure 1A, B), but not other eukaryotic lineages, in agreement with previous studies [31], [32]. All members of the BSU1-like family including the single example contained in each *Plasmodium spp* share a distinctive conserved protein architecture, with N-terminal kelch repeats followed by the phosphatase domain.

*P. berghei* PPKL contains five complete and one truncated kelch repeat (Figure 1A), a configuration which is conserved in most identified orthologues. Phylogenetic trees and identity matrices of individual kelch domains showed that the fourth domain has the highest identity between species, while the sixth was the least similar (Figure S1). The phosphatase domain of *P. berghei* PPKL shows the greatest similarity to type 1 and 2A protein phosphatases. All the important signature motifs of the serine/threonine phosphatase (STP) family are present (Figure S2); they form the active site, and are known to have a role in metal ion binding (GDxHG, GDxVDRG, and GNHE) [39]. The binding site for the inhibitor microcystin is conserved, but some of the residues important for docking the PP1 inhibitor Inhibitor-2 and the PPP inhibitor okadaic acid are not conserved, making it difficult to predict *in silico* the inhibitor specificity of this enzyme. Additionally, we identified 4 conserved insertions (Figure S2I – IV) in the catalytic domain, which are only present in *Apicomplexa*, suggesting a specific role for these sequences in this group.

### PPKL has protein phosphatase enzyme activity, and is highly expressed in female gametocytes and ookinetes

**Figure 2:**
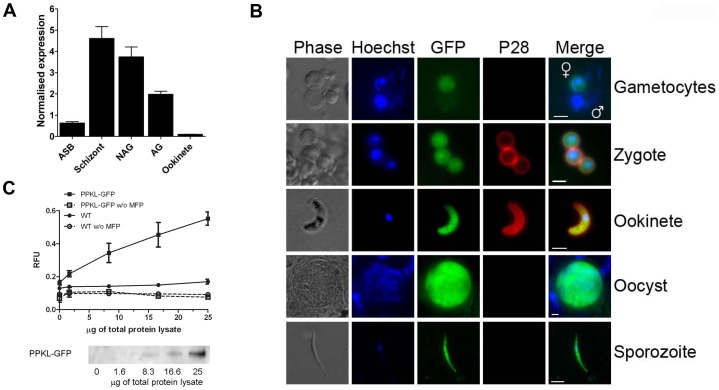
Expression of PPKL-GFP during the *Plasmodium* life-cycle in the mosquito and PPKL-GFP phosphatase activity. A. Wild-type mRNA expression of *ppkl* relative to *hsp70* and *arginyl-tRNA synthetase* as endogenous controls (ΔΔCt method). Error bar = ±SEM, *n = *3 from three independent experiments. ASB = asexual stages in blood; NAG = non-activated gametocytes; AG = activated gametocytes. B. PPKL-GFP expression in transgenic parasites during the sexual phase of the life-cycle and sporogony. Bar = 5 µm. Note that in gametocytes (the male gametocyte contains the enlarged nucleus) only the female expresses PPKL-GFP. C. Upper panel: phosphatase activity in immunoprecipitated lysates of PPKL-GFP and WT parasite lines in the presence or absence of MFP substrate. Error bar = ±SEM, *n = *6. Lower panel: anti-GFP Western blot showing amounts of PPKL-GFP retained from the corresponding lysate.

Transcriptomic and proteomic studies in *Plasmodium* [36], [37] have suggested that *ppkl* transcripts and PPKL protein are present in gametocytes and ookinetes only, respectively, although PPKL is not present in the proteome of sex-specific gametocytes [18]. To confirm this, we analysed *ppkl* mRNA expression by qRT-PCR and PPKL protein expression and localisation by generation of a C-terminal green fluorescent protein (GFP) fusion protein of endogenous *ppkl* (PBANKA_132950) using a single crossover recombination strategy (Figure S3A–E). *ppkl* mRNA is expressed in asexual blood stages, gametocytes and ookinetes, with the highest expression in schizonts (Figure 2A) relative to *hsp70* and *arginyl-tRNA synthetase* genes used as controls in this assay. The intensity of PPKL-GFP fluorescence in sexual stages was highest in the nuclear and cytoplasmic compartments of female gametocytes and zygotes, and in the apical cytoplasm of ookinetes. The protein was also detected in oocysts and sporozoites, but was not observed in activated microgametocytes or microgametes (Figure 2B). These data suggest that in sexual stages PPKL is female-specific and has a role during sexual development.

We also investigated whether PPKL is an active phosphatase in *P. berghei*. Using 3-O-methylfluorescein phosphate (MFP) as a substrate, we found that PPKL-GFP immunoprecipitated with GFP-TRAP beads from parasite lysates had phosphatase activity proportional to the amount of lysate in the assay; whereas the WT parasite lysate controls produced much lower fluorescence levels (Figure 2C). The residual fluorescence observed in the WT lysate compared to the corresponding control without MFP substrate might be due to endogenous phosphatase activity non-specifically pulled-down by the GFP-TRAP beads. Our results therefore indicate that PPKL has protein phosphatase activity, correlating with its orthologue in *Arabidopsis*, BSU1 [34].

### An absence of PPKL results in abnormal ookinete morphology and is contributed by the female gamete

**Figure 3:**
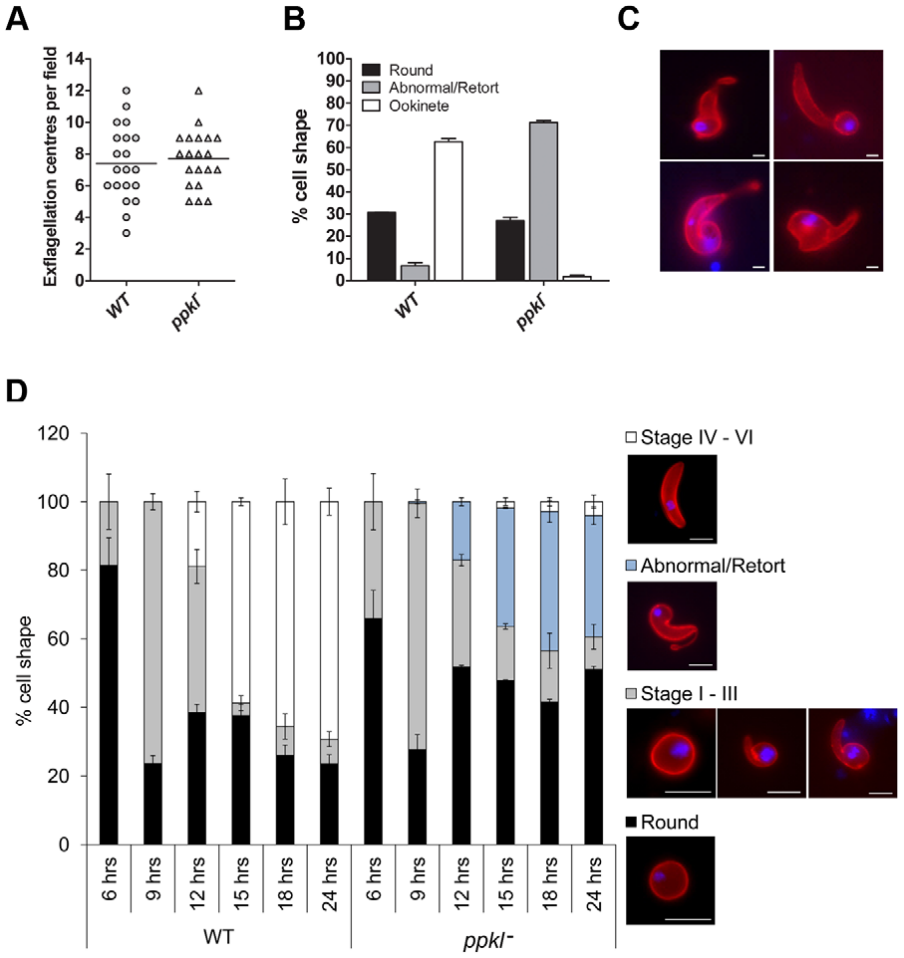
Gametocyte activation and ookinete differentiation of *ppkl^−^* mutants. A. Exflagellation of activated male gametocytes of *ppkl^−^* mutants compared to wild-type (bar = arithmetic mean, *n = *20, ×40 magnification). B. Ookinete conversion in wild-type and *ppkl^−^* parasites. Conversion rate is the percentage of P28 (ookinete surface protein)-positive parasites that had differentiated into ‘banana-shaped’ ookinetes (error bar = ±SD; *n* = 3). C. Different morphological shapes of *ppkl^−^* abnormal retorts as assessed by P28 staining. Bar = 1 µm. D. Time-course analysis of ookinete differentiation in wild-type and *ppkl^−^* parasites. Morphologies were grouped into four categories: unfertilized macrogametes/zygotes (round – black bars); stages I–III (gray bars); abnormal/retorts (light blue bars); and stages IV–VI (white bars). Error bar = ±SD, *n = *3. Panels to the right of the graph show ookinetes at different stages of maturity immunolabelled with the anti-P28 Cy3-conjugated 13.1 antibody used for scoring. Bar = 5 µm.

To examine the function of PPKL during the *Plasmodium* life-cycle, we used a double homologous recombination strategy to replace the endogenous *ppkl* gene with a *dhfr*/*ts* selectable marker from *Toxoplasma gondii* (Figure S4A–E). Analysis of two *ppkl* deletion mutant clones from two independent transfections, named *ppkl^−^* cl3 and *ppkl^−^* cl9 identified no phenotypic changes during asexual proliferation in terms of parasite growth, erythrocyte invasion or morphology and no effect on sexual stage cell development (gametocytogenesis), as assessed on blood smears (data not shown). Gametogenesis in the activated microgametocyte was also comparable to wild-type controls (Figure 3A). However, analysis of *in vitro* cultures for 18–24 h to monitor differentiation into ookinete stages [40] showed that cultures of *ppkl^−^* mutants were dominated by grossly abnormal retort forms [41] (Figure 3B, C, S5C). As a result of this observation, we performed a time-course analysis of ookinete differentiation over 6, 9, 12, 15, 18 and 24 h. Development of the zygote through stages I–III of ookinete maturity (0–9 h post-fertilisation) [41] was indistinguishable in the *ppkl^−^* mutants compared to wild-type. Wild-type controls showed normal progression and maturation to stage IV (at 12 h), with 69% of all macrogamete-derived parasites progressing to stage VI 24 h post-fertilisation (Figure 3D). In contrast, the majority of *ppkl^−^* mutants did not progress from stage III to stage IV, but produced a high proportion of abnormal retort forms 24 h post fertilisation (35% of the total population) with only 4% progressing to stage IV–VI. This suggests that PPKL is essential for maturation, differentiation and morphological development of ookinetes from stage III to stage IV.

**Figure 4:**
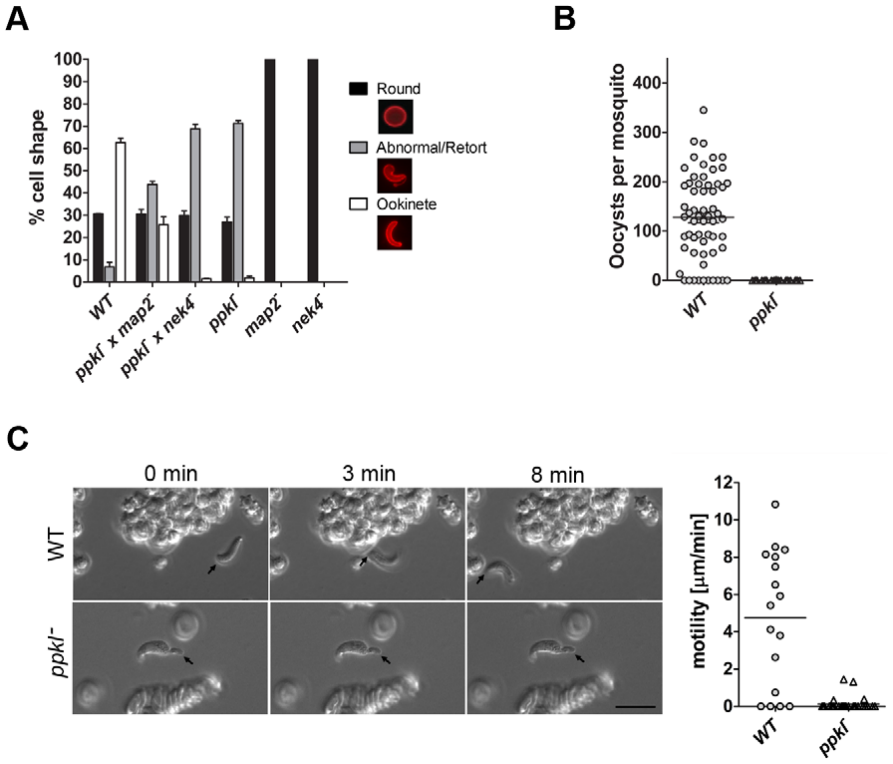
Genetic crossing, *in vivo* transmission and gliding motility of *ppkl^−^* parasites. A. Ookinete conversion after crossing *ppkl* ^−^ mutants with female-defective (*nek4^−^*) or male-defective (*map2^−^*) mutants. Wild-type parasites were used as a control. Bar graph shows the percentage of round P28-positive parasites that had converted into elongated ookinetes and retorts (error bar = ±SD; *n* = 3). B. Average number of oocysts per mosquito gut (day 14 post-infection; bar = arithmetic mean; *n = *60 of wild-type or *ppkl^−^* infected mosquitoes from three independent experiments). Overall infection prevalence was 85% for wild-type and 0% for *ppkl^−^*. C. Representative frames from time-lapse videos of a wild-type ookinete (upper panels) and *ppkl^−^* abnormal retort (lower panels) in Matrigel. Black arrow indicates the apical end of the ookinete/abnormal retort. Bar = 10 µm. Speed of individual wild-type ookinetes or *ppkl^−^* abnormal retorts from 24 h ookinete cultures measured over a 10 min period is shown in the dot plot. Bar = arithmetic mean; *n = *17 for wild-type and 28 for *ppkl^−^* lines.

We next examined whether the defect was sex-specific by performing genetic crosses between *ppkl^−^* parasites and lines deficient in either male (*map2^−^*) or female (*nek4^−^*) gametes [14], [40]. Cross-fertilization with *nek4^−^* parasites did not rescue the phenotype, whereas crossing with *map2^−^* gametocytes resulted in 26% of all macrogamete-derived parasites progressing to stage VI, revealing that the requirement for the phosphatase is inherited through the female line (Figure 4A).

### PPKL is essential for gliding motility and mosquito invasion

To substantiate the *in vitro* findings, parasites in mice infected with either wild-type or *ppkl^−^* gametocytes were fed to mosquitoes to analyse oocyst development. Wild-type oocysts developed normally; whereas no oocysts were found in the guts of mosquitoes fed on *ppkl^−^* parasites when analysed 14 and 21 days after feeding (Figure 4B). This result confirms that PPKL is vital to ookinete development and that oocyst formation is completely blocked in the *ppkl^−^* parasites.

Due to the ablation of oocyst development in our *in vivo* study (Figure 4B), as well as defects in the morphology and maturation of *ppkl^−^* mutants, we analysed gliding motility of wild-type and *ppkl^−^* parasites by embedding cultured ookinetes in dilute Matrigel and using time-lapse video microscopy to quantify gliding movement [20]. Using this method, we found that wild-type parasites followed a characteristic helical gliding motion with an average speed of 4.74 µm/min (Figure 4C and Video S1), in close agreement with previous studies [20]. Strikingly, gliding motility in *ppkl^−^* retorts was significantly reduced compared to wild-type parasites (0.13 µm/min; *p*<0.001), although occasional limited forward motion and “flexing” at the apical end was observed (Figure 4C and Video S2).

To assess whether the ablation of oocyst development in *ppkl^−^* mutant parasites was also due to a defect in invasion of the mid-gut epithelium, we bypassed the gut barrier by injecting *ppkl^−^* parasites directly into the haemocoel of *A. stephensi* mosquitoes and analyzed salivary gland invasion 20 days post-injection [42]. Using this method, we found that *ppkl^−^* parasites were able to form viable sporozoites, which could migrate to the salivary gland and actively invade (Table 1). Onward transmission experiments via a mosquito biting resulted in infection in mice with both wild-type and *ppkl^−^* lines. Subsequent analysis of parasites recovered from these lines and cultured in ookinete medium for 24 h confirmed the knockout phenotype in the *ppkl^−^* lines. This strongly suggests that sporozoites of *ppkl^−^* mutants are able to migrate to and invade the salivary glands, as well as undergo exo- and intra-erythrocytic proliferation, but the abnormal retorts of *ppkl^−^* are not able to penetrate the epithelial lining of the mosquito mid-gut.

**Table 1:** Sporozoite infectivity in ookinetes directly injected into the haemocoel.

Experiment	Parasite line	Number of salivary gland sporozoites/mosquito	Biteback (Ψ)	First day of detectable blood stage infection
1	Wild type	1840	2/2 (14/23)	4 dpi
	*ppkl^−^*	147	1/2 (11/8)	6 dpi
2	Wild type	29′321	2/2 (31/23)	4 dpi
	*ppkl^−^*	20′500	2/2 (27/23)	4 dpi

Ψ = number of mosquitoes fed.

dpi = days post-infection.

### *ppkl^−^* parasites show defects in ookinete apical end integrity

To examine whether the morphology of the *ppkl^−^* mutant retorts could be linked to their inability to glide and invade, we performed ultrastructure analyses using transmission electron microscopy (TEM).

**Figure 5:**
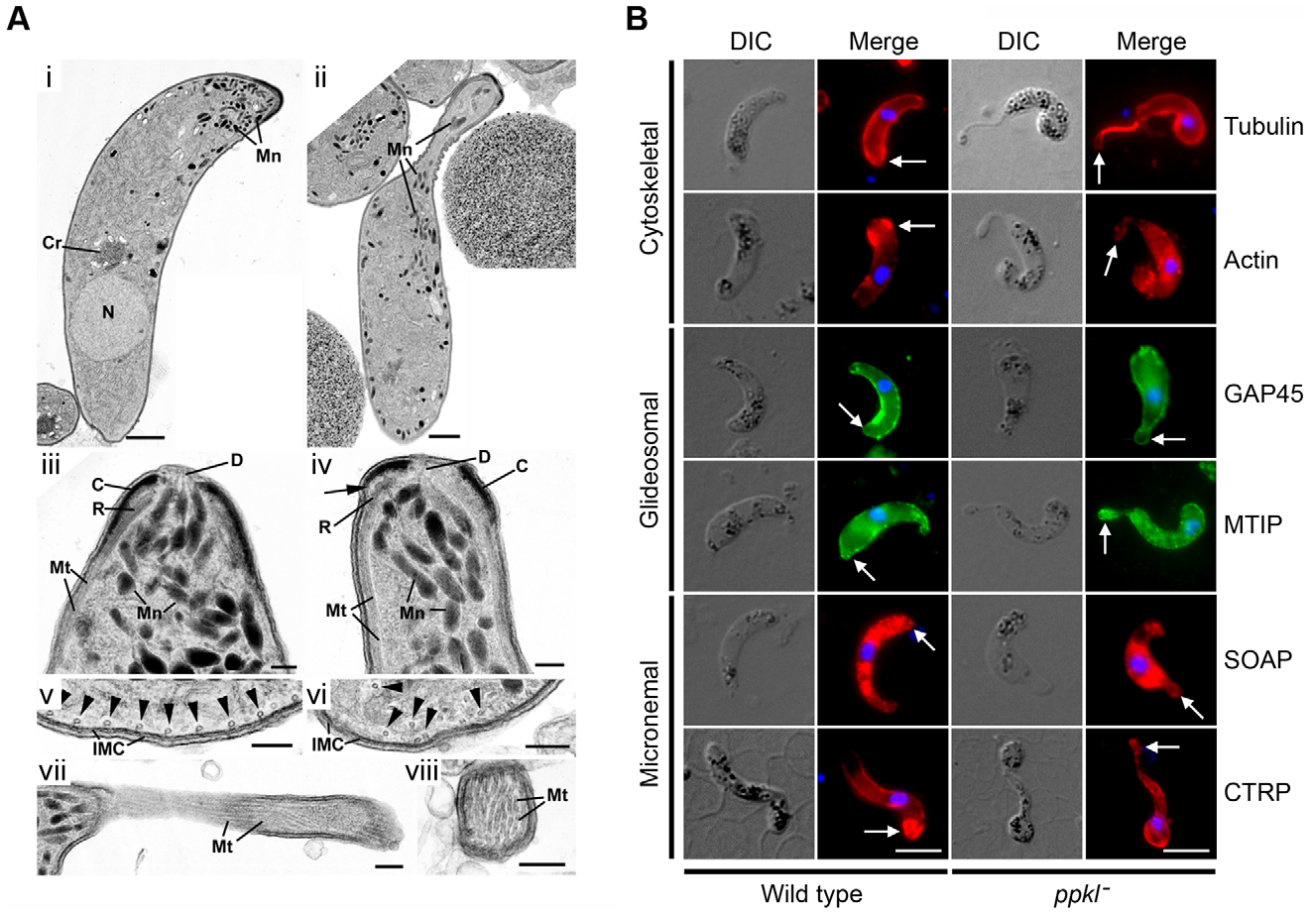
Ultrastructure analysis and indirect immunofluorescence of ookinetes. A. (i) Transmission electron micrograph (TEM) of a longitudinal section though a wild-type ookinete showing the conical apical end. The cytoplasm contains a number of apically located micronemes (**Mn**), a more posteriorly located nucleus (**N**) and a central crystalloid body (**Cr**). Bar = 1 µm. (ii) TEM of a longitudinal section though an ookinete of the *ppkl^−^* mutant showing partial collapse and elongation of the apical end. Within the cytoplasm, the micronemes (**Mn**) are more randomly distributed. Bar = 1 µm. (iii) Enlargement of the anterior of a wild-type ookinete illustrating the complex nature of the apical end consisting of a conical shaped electron dense collar (**C**) with a central aperture. Underlying the collar and in contact with it is a less electron dense ring (**R**) that is also in contact with longitudinally running sub-pellicular microtubules (**Mt**). Micronemes (**Mn**) are located within the cytoplasm with small ducts (**D**) running though the apical aperture to the plasmlemma. Bar = 100 nm. (iv) Detail of the anterior of a *ppkl^−^* ookinete showing a less conical shape associated with a reduction in the length of the electron dense collar (**C**) and some separation (arrow) from the electron lucent ring (**R**) with associated microtubules (**Mt**). The few micronemes (**Mn**) present showed ducts (**D**) running to the anterior. Bar = 100 nm. (v) Part of a cross section though the anterior end of a wild-type ookinete showing the specific inter-relationship between the microtubules (arrowheads) and the inner membrane complex (**IMC**). Bar = 100 nm. (vi) Cross-section though a *ppkl^−^* ookinete illustrating microtubules (arrowheads) within the cytoplasm that have lost contact with the IMC. Bar = 100 nm. (vii) Longitudinal section though the apical end of a severely affected *ppkl^−^* ookinete showing the collapsed and elongate neck-like structure containing longitudinally running microtubules (**Mt**). Bar = 100 nm. (viii) Cross section though the collapsed neck region showing it to consist of microtubules. Bar = 100 nm. B. Indirect immunofluorescence detection of a number of cytoskeletal, glideosomal and micronemal proteins in wild-type ookinetes and *ppkl^−^* abnormal retorts (24 h) post-gametocyte activation. Abnormally intense tubulin staining was observed at the ookinetes apical end (red). Motor protein GAP45 and MTIP distributions (green) did not show any difference in the mutant compared to wild-type. The apical localisation of actin and micronemal CTRP was more diffuse throughout the cell body (red) in the *ppkl^−^* mutant. However, the usual diffuse intracellular staining of SOAP showed no obvious abnormal pattern in the mutant. The nucleus was counter-stained using DAPI (blue). Arrows indicate the apical end of the ookinete. Bar = 5 µm.

Significant structural differences were detected between wild-type and *ppkl^−^* mutant parasites at the apical end of the ookinete. Wild-type ookinetes had a very uniform appearance with a conical shaped apical end (Figures 5Ai), with unique and complex structures [43] consisting of an enclosing plasmalemma, beneath which is a conical electron dense collar with a central aperture and in firm contact with the IMC (Figure 5Aiii, S5A). Beneath the collar, but in close contact with it, is a second somewhat smaller and less electron dense layer termed the apical ring (Figure 5Aiii, S5A), which is subtended by the sub-pellicular microtubules. The apical end also has a large number of micronemes with fine ducts running through the aperture in the collar to the apical plasmalemma (Figure 5Aiii, S5A). In contrast, *ppkl^−^* retort forms showed marked variation in shape with clear constriction and elongation of the apical end (Figure 5Aii, 5Avii and S5B). Detailed examination of the apical end showed structural differences from the wild-type, namely: the electron dense collar's reduced length (Figure 5Aiv, S5B); the apical ring appeared reduced in size compared to the wild-type, resembling a series of small vesicles and some loss of connection to the collar (Figure 5Aiv); the microtubules run longitudinally from the ring, but while they were closely associated with the IMC in the wild-type parasite (Figure 5Av), they were more disorganised in the mutant and the electron dense links between the microtubules and the IMC were not found (Figure 5Avi); in severe cases, collapse of the apex resulted in groups of tubules within a neck-like region not associated with the IMC (Figure 5Avii, viii). Due to the collapse of the apex, fewer micronemes were located in the apical region, but those present appeared normal with ducts running to the apex (Figure 5Aiv, S5B). The other cytoplasmic and nuclear features were similar in both wild-type and mutant parasites (Figure 5Ai, ii). In summary, there is a loss of apical integrity due to defects in the collar and the attachment between the microtubules and IMC possibly resulting in an immotile parasite.

Indirect immunofluorescence analysis using antibodies for the cytoskeletal proteins tubulin and actin showed mislocalisation with intense atypical tubulin staining at the apical end of the *ppkl^−^* abnormal retort as well as loss of the normal apical staining for actin. While the motor complex proteins GAP45 and MTIP showed no obvious abnormal pattern in *ppkl^−^* lines, micronemal CTRP showed less distinct apical staining and is more distributed throughout the body of the retort. This may be due to the structural and cytoskeletal changes at the apical end. However, no change in the normally diffuse intracellular distribution of the microneme-associated protein SOAP was observed (Figure 5B).

### Expression of genes essential to zygote development is differentially regulated in PPKL mutants

**Figure 6:**
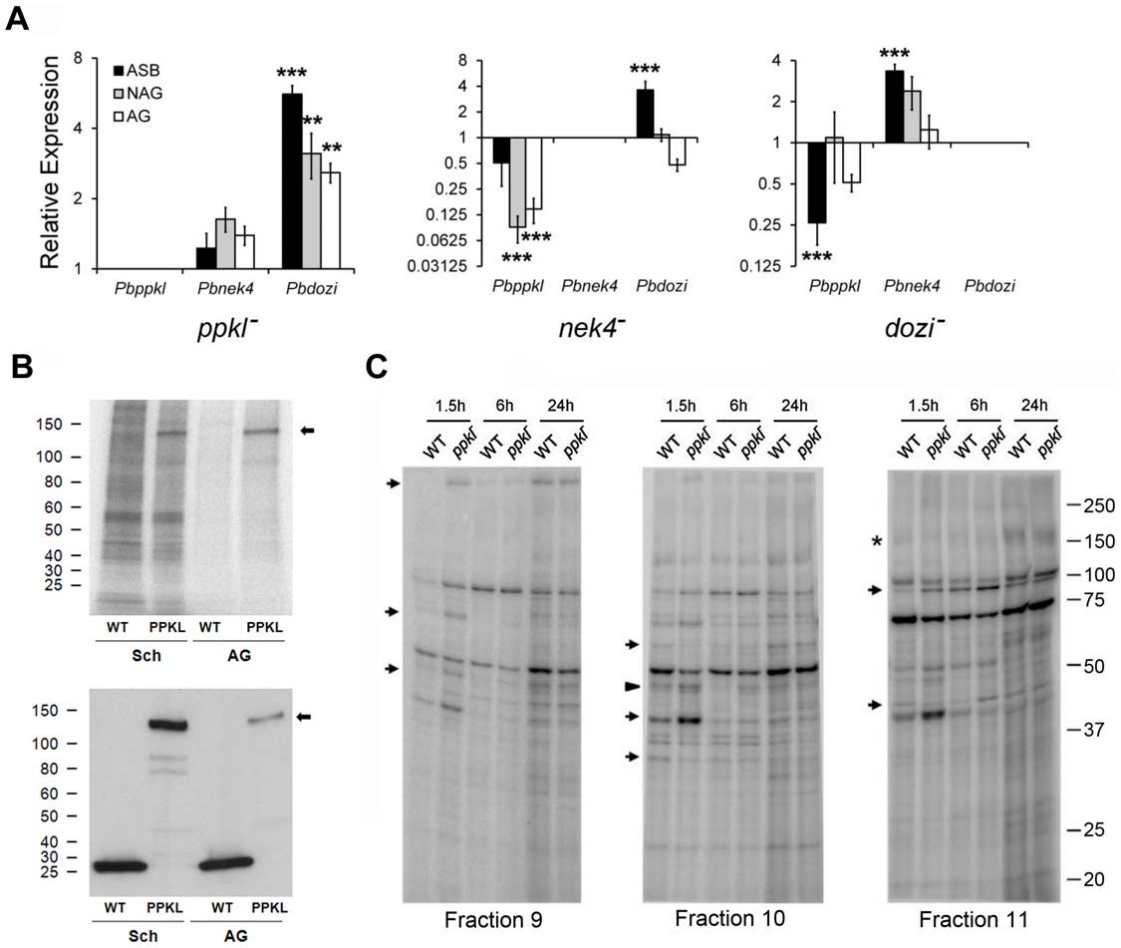
Differential transcript and phosphorylation levels in *ppkl^−^* and mutants of genes essential for zygote development. A. Relative expression of *ppkl*, *nek4* and *dozi* in *ppkl^−^, nek4^−^* and *dozi^−^* mutant parasites compared to wild-type controls (Pfaffl method). Error bar = ±SEM, *n = *3 from three independent experiments. ***p≤0.001; **p≤0.01. ASB = asexual blood stages; NAG = non-activated gametocytes; AG = activated gametocytes. B. Upper panel: autoradiograph showing phosphorylation in lysates of schizonts and activated gametocytes from WT-GFP and PPKL-GFP parasite lines. Lower panel: corresponding Western blot using anti-GFP antibody. Sch = schizonts; AG = activated gametocytes. C. Autoradiograph from three fractions showing alterations in global phosphorylation 1.5, 6 and 24 h post-activation of wild-type and *ppkl^−^* gametocytes. Differential phosphorylation in *ppkl^−^* compared to wild-type 1.5 h after gametocyte activation is represented by arrows, 6 h after gametocyte activation by an arrowhead and 24 h after gametocyte activation by an asterisk. Representative radiographs of three independent experiments are shown.

The protein kinase NEK4 and the DDX6-class RNA helicase DOZI are essential to female/zygote development [14], [18], [44]. To ascertain whether PPKL is regulated by either of these, we analysed mRNA expression of the phosphatase in mutants of NEK4 and DOZI by qRT-PCR. Expression of *ppkl* compared to wild-type was only significantly altered in both non-activated and activated gametocytes of the *nek4^−^* mutant (*p*<0.001 for both). In *ppkl^−^* mutants, transcript levels of *nek4* compared to wild-type controls were indistinguishable in both total asexual blood stages and non-activated and activated gametocytes (Figure 6A). However, *dozi* was significantly up-regulated compared to wild-type (*p*<0.01 for all stages analysed), suggesting that PPKL could be involved in the function of this RNA helicase.

### PPKL is hyper-phosphorylated in activated gametocytes compared to schizonts

As studies in *Arabidopsis thaliana* have shown that the activity of BSU1 is regulated by phosphorylation [45], we investigated whether PPKL was phosphorylated in *P. berghei* using PPKL-GFP parasites metabolically labelled for 30 min with ^32^P-orthophosphate, lysed and immunoprecipitated with GFP-TRAP to assess PPKL phosphorylation *in vivo*. Activated gametocytes showed a higher level of phosphorylation compared to schizonts (7.6 times) as assessed by measuring the ratio of the intensity of the PPKL phosphorylation signal on the autoradiograph to the intensity of the PPKL protein band on the Western blot (Figure 6C, arrow).

### Global phosphorylation is altered in *ppkl^−^* mutants

As PPKL is essential for ookinete maturation, we assessed the impact of *ppkl* deletion on protein phosphorylation during ookinete development. This was achieved by analysing global phosphorylation profiles of wild-type and *ppkl^−^* parasites 1.5, 6 and 24 h post gametocyte activation (pga) using a metabolic labelling technique [46]. Three representative fractions (9–11), in which significant differences in phosphorylation were observed are shown in Figure 6C. Equal protein loading was assessed by Coomassie blue staining (data not shown). While the majority of the phosphorylated proteins remained unchanged in wild-type versus *ppkl^−^* lysates, as early as 1.5 h pga (Figure 6C, arrows), and also later in zygote development (6 h) (Figure 6C, arrowhead) and 24 h pga (Figure 6C, asterisk), we observed several specific changes, including both increases and decreases in phosphorylation levels. These data suggest that although PPKL is involved in the regulation of the phosphorylation status of specific proteins throughout ookinete development, the largest impact of PPKL on protein phosphorylation occurs early (1.5 h) after gametocyte activation.

## Discussion

Reversible phosphorylation is a major regulator for many cellular processes. The kinases are well recognised as important signalling molecules and drug targets in various diseases, but phosphatases have been neglected and their roles remained elusive until recently [32], [47]. Although recent systematic functional analyses of the *Plasmodium* kinome have identified a number of potential drug targets [9], [10], studies on phosphatases in *Plasmodium* have been mostly limited to biochemical studies. Recently however, the total protein phosphatome of *P. falciparum* was published and showed that the genome of this malaria parasite codes for one of the smallest known eukaryotic phosphatomes, comprising 27 phosphatase sequences [28]. We have shown that the unique phosphatase PPKL is encoded by a single-copy gene belonging to a robust family that is only detected in *Viridiplantae* and *Alveolata*, confirming previous studies [31], [32]. Analysis of wild-type mRNA transcription and protein expression confirmed the findings of previous global studies [36], [37], suggesting that PPKL is expressed during sexual development. Furthermore, we have also shown that PPKL is an active phosphatase, which in gametocyte/gamete stages is female-specific and in the ookinete is found preferentially at the apical end. However, the localisation is not uniquely nuclear but is also cytosolic, contrasting the exclusively nuclear localisation of the *Arabidopsis* BSU1 phosphatase [34].

Our functional studies have shown that the only point of essentiality for PPKL in the malaria parasite is ookinete differentiation, particularly in the latter stages when the machinery for motility and invasion is formed. Zygote development is normal in *ppkl^−^* mutants, but ookinete differentiation is grossly impaired, producing a parasite population dominated by retorts with abnormal morphology and varying degrees of structural deformities. By using a motility assay we show here that the gliding motility of *ppkl^−^* abnormal retorts is virtually abolished. The data suggest that apical structure and organelle distribution are determined at the early female/zygote stage. We also show that this defect is inherited through the female line. Previous studies have shown that many defects at the zygote/ookinete stage are carried through maternal inheritance of a number of ookinete specific molecules [42]. In addition, the haemocoel infection experiment clearly suggests that this impairment is restricted to the ookinete stage since by-passing the mid-gut barrier by direct injection of mutant retorts into the haemocoel allowed oocyst formation and the production of invasive sporozoites that could cause blood stage infection after a mosquito bite-back. However, the abnormality persisted at the subsequent ookinete stage in these *ppkl^−^* parasites.

The ookinete is morphologically and biochemically distinct from sexual stage gametocytes and zygotes, as well as the later oocyst and sporozoite stages. It is also the only motile and invasive stage that is formed extracellularly, does not require invasion of a new host cell as it moves between the mosquito gut cells in traversing to the basal surface and also develops extracellularly into the next stage. Ultrastructure analyses using TEM revealed severe structural abnormalities in the apical end of the mutant retort forms, particularly that of microtubule organisation and association with the IMC, as well as a reduction in the length of the apical ring and distinct shortening of the apical collar. The apical complex of the ookinete is unique amongst the invasive *Plasmodium* stages. In particular, the distinctive ookinete morphology is maintained through an array of microtubules, the subpellicular network [22], and a lattice of intermediate filaments. These are enclosed by the pellicle consisting of the plasmalemma and two underlying closely associated unit membranes formed from flatten vacuoles and referred to as the IMC. The structural abnormalities seen in the microtubular association with the IMC, as well as the mislocalisation of cytoskeletal proteins in *ppkl^−^* lines could explain the distinctive morphology of the mutant. It would also explain why no *ppkl^−^*-associated defects are observed in the rest of the life-cycle since the unique apical complex of the ookinete is absent in both merozoites and sporozoites.

Immunofluorescence studies with antibodies to the microneme marker CTRP suggested that in *ppkl^−^* lines CTRP is distributed throughout the parasite body and the apical end localisation as seen in the wild-type parasite is not observed. It is important to note however, that even though CTRP and SOAP are both microneme-associated proteins, the localisation we see in our wild-type parasites is consistent with previous studies [26], [48], with CTRP showing intense apical staining and SOAP localisation more diffuse throughout the cell body. The altered distribution of CTRP in *ppkl^−^* lines could more likely be due to the structural abnormality at the apical end, since we still observe a high concentration of apical micronemes in the mutants. Although this may explain their inability to move and invade the gut wall, it has been shown previously in *misfit* disruption mutants [49] that even in the absence of micronemes, parasites could still form oocysts. Nevertheless, this particular defect in MISFIT is quite different to *ppkl^−^* because it shows paternal inheritance and a range of distinct molecular defects. The location of markers of the motor complex such as GAP45 and MTIP did not show any marked difference between wild-type and *ppkl* ^−^ parasites. However, the mislocalisation of the cytoskeletal protein tubulin and of actin in *ppkl^−^* parasites is consistent with the structural defects identified in the ultrastructure studies. In mammalian systems protein phosphatases are important in microtubule formation and are regulated during the cell cycle [50]; it is therefore possible that PPKL has a direct role in microtubule distribution, consistent with the aberrant morphology of the *ppkl* ^−^ retorts.

Cellular processes are regulated through complex signalling networks, although very little is known in *Plasmodium*. Studies of kinases have demonstrated the importance and specificity of protein phosphorylation at every stage of the *Plasmodium* life-cycle. For example, a number of kinases are implicated in male gamete (CDPK4, SRPK, MAP2), female gamete (NEK2, NEK4), zygote (NEK2, NEK4), and ookinete (PK7, GAK) development [10], [51]. We have shown that *ppkl* transcripts are down-regulated in *nek4^−^* mutants. Furthermore, we have also shown that PPKL is phosphorylated in both schizonts and activated gametocytes, confirming phosphoproteomic studies indicating that PPKL is itself phosphorylated [52] and suggesting that its activity or interactions with other proteins may also be regulated by phosphorylation. As a precedent, phosphorylation of the *Arabidopsis* PPKL orthologue, BSU1, by the constitutive differential growth 1 (CDG1) kinase results in its activation and the subsequent dephosphorylation of BIN2 [45], suggesting a fine balance between phosphorylation/dephosphorylation pathways in signalling in plants [53]. Whether such a mechanism operates in *Plasmodium* is to be elucidated in future studies.

The global protein phosphorylation profile during ookinete development of the *ppkl^−^* mutant is evidently different from that of wild-type parasites. Most changes were seen within 1.5 h of gametocyte activation and zygote formation, suggesting that the function of PPKL is crucial at the early zygote stage even though the phenotype is observed at the ookinete stage. For example, the molecular blueprint for apical end formation may be established following fertilization in the early zygote stage. One intriguing observation is that the qRT-PCR analyses revealed that transcripts of *dozi* (which is involved in mRNA processing) [44] are significantly up-regulated in *ppkl^−^* lines. This might suggest that in the absence of the phosphatase the modulation of DOZI is perturbed and this could directly affect the process of translational repression for many proteins in early zygote development. Previous studies have identified IMC-associated proteins that are vital for maintenance of ookinete morphology and virulence, namely IMC1b and IMC1h (or Alv3) [54]–[56]. Disruption and deletion mutants of the nucleotide cyclase guanylyl cyclase β (GCβ) and the cyclic nucleotide degrading phosphodiesterase δ (PDEδ), respectively, showed unregulated signalling via cGMP resulting in defective ookinete development and gliding motility [20], [57]. Moreover, mutants of CDPK3 [21], micronemal proteins including CTRP and SOAP [25], [26], PPL3 and 5 [58], and other molecules such as PSOP2 and PSOP7 show severe defects in ookinete formation and gliding motility, respectively [42]. Whether PPKL interacts with any of these vital regulators of ookinete biology will be dissected in future studies.

In summary, this is the first functional analysis, to our knowledge, of a protein phosphatase in *Plasmodium* and demonstrates that like the kinases, a phosphatase is also involved in a regulatory pathway in a stage-specific and essential manner.

## Materials and Methods

### Ethics statement

All animal work has passed an ethical review process and was approved by the United Kingdom Home Office. Work was carried out in accordance with the United Kingdom ‘Animals (Scientific Procedures) Act 1986’ and in compliance with ‘European Directive 86/609/EEC’ for the protection of animals used for experimental purposes. The permit number for the project licence is 40/3344.

### Animals

Tuck-Ordinary (TO) (Harlan) outbred mice were used for all experiments except for mosquito “bite-back” infections of mice, where C57/Bl6 mice were used.

### Generation of transgenic parasites

The targeting vector for *ppkl* was constructed using the pBS-DHFR plasmid, which contains polylinker sites flanking a *Toxoplasma gondii dhfr/ts* expression cassette conveying resistance to pyrimethamine, as described previously [10]. PCR primers P0011P (5′-CCCCGGGCCCCATGTTTTATATTGTGTTTTGGC-3′) and P0012P (5′-GGGGAAGCTTCAAACATTCGTTTCTTTAAATGATCC-3′) were used to generate a 788 bp fragment of 5′ upstream sequence of *ppkl* from genomic DNA, which was inserted into *Apa*I and *Hind*III restriction sites upstream of the *dhfr/ts* cassette of pBS-DHFR. A 694 bp fragment generated with primers P0013P (5′-CCCCGAATTCCCACCAACCCCACCAAGAAGTCAACCG-3′) and P0014P (5′-GGGGTCTAGACCGGCAAATTGATGAAATCGC-3′) from the 3′ flanking region of *ppkl* was then inserted downstream of the *dhfr/ts* cassette using *Eco*RI and *Xba*I restriction sites. The linear targeting sequence was released using *Apa*I/*Xba*I. For GFP-tagging by single homologous recombination, a 1047 bp region of *ppkl* starting 1693 bp downstream of the ATG start codon and omitting the stop codon was amplified using primers P1tag F (5′-CCCCGGTACCGAGCTCCGATAAAAATATATGGTGATATAC-3′) and P1tag R (5′-CCCCGGGCCCTGGAGCCCCATAATTTAATTCTCTC-3′), producing an amplicon 1047 bp in length. This was inserted upstream of the *gfp* sequence in the p277 vector using *Kpn*I and *Apa*I restriction sites. The p277 vector contains the human *dhfr* cassette, also conveying resistance to pyrimethamine. Before transfection, the sequence was linearized using *Bgl*II and *P. berghei* ANKA line 2.34 was then transfected by electroporation [59]. Briefly, electroporated parasites were mixed immediately with 200 µl of reticulocyte-rich blood from a phenylhydrazine (Sigma) treated, naïve mouse, incubated at 37°C for 20 min and then injected intraperitoneally. From day 1 post infection pyrimethamine (70 µg/ml) (Sigma) was supplied in the drinking water for four days. Mice were monitored for 15 days and drug selection was repeated after passage to a second mouse. Resistant parasites were then used for cloning by limiting dilution and subsequent genotyping.

### Genotypic analysis of mutants

Chromosomes of wild type and gene knockout parasites were separated by pulsed field gel electrophoresis (PFGE) on a CHEF DR III (BioRad) using a linear ramp of 60–500 s for 72 h at 4 V/cm. Gels were blotted and hybridized with a probe recognizing both the resistance cassette in the targeting vector and, more weakly, the 3′UTR of the *P. berghei dhfr/ts* locus on chromosome 7. For the gene knockout parasites, two diagnostic PCR reactions were used as illustrated in Figure S4. Primer 1 (INT P1P, 5′- CGCATAAAGTGTTGCATTATATAAATTACAC-3′) and primer 2 (ol248, 5′-GATGTGTTATGTGATTAATTCATACAC-3′) were used to determine successful integration of the selectable marker at the targeted locus. Primers 3 (P1 KO1P, 5′- CACCCCCAGAAGCTAGATATCAACATACTTGCG-3′) and 4 (P1 KO2P, 5′- GAACTAGGTGAATCGAGCATATTTCTGTAG-3′) were used to verify deletion of the gene. Having confirmed integration, genomic DNA from wild type and mutant parasites was digested with *EcoR*I and the fragments were separated on a 0.8% agarose gel, blotted onto a nylon membrane (GE Healthcare), and probed with a PCR fragment homologous to the *P. berghei* genomic DNA just outside of the targeted region.

For the C-fusion GFP tagging parasites, one diagnostic PCR reaction was also used as illustrated in Figure S3. Primer 1 (INT P1, 5′-GGTCAAATGTATCTATATTATGTTC-3′) and primer 2 (ol492, 5′- ACGCTGAACTTGTGGCCG-3′) were used to determine correct integration of the *gfp* sequence at the targeted locus. Having confirmed correct integration, genomic DNA from wild type and transgenic parasites was digested with *EcoR*I and the fragments were separated on a 0.8% agarose gel, blotted onto a nylon membrane, and probed with a PCR fragment homologous to the *P. berghei* genomic *ppkl* sequence using the Amersham ECL Direct Nucleic Acid Labelling and Detection kit (GE Healthcare). Parasites were also visualized on a Zeiss AxioImager M2 (Carl Zeiss, Inc) microscope fitted with an AxioCam ICc1 digital camera (Carl Zeiss, Inc) and analysed by Western blot to confirm GFP expression.

### Phenotypic analysis

Infections for phenotype screens were initiated by intraperitoneal injection of infected blood containing 5×10^6^ parasites into mice pre-treated with 0.2 ml of 6 mg/ml phenylhydrazine in PBS injected intraperitoneally to induce reticulocytosis 3 days prior to infection. Asexual stages and gametocyte production were monitored on Giemsa-stained blood films.

Exflagellation was examined on day 4–5 post infection. 10 µl of gametocyte-infected blood were obtained from the tail with a heparinized pipette tip and mixed immediately with 40 µl of ookinete culture medium (RPMI1640 containing 25 mM HEPES, 20% fetal bovine serum, 10 mM sodium bicarbonate, 50 µM xanthurenic acid at pH 7.6). The mixture was placed under a Vaseline-coated cover slip and 15 min later exflagellation centres were counted by phase contrast microscopy in 12–15 fields of view using a 63× objective and 10× ocular lens. Ookinete formation was assessed the next day. 10 µl of infected tail blood were obtained as above, mixed immediately with 40 µl ookinete culture medium, and incubated for 2 h at 20°C to allow completion of gametogenesis and fertilization. Each culture was then diluted with 0.45 ml of ookinete medium and incubated at 20°C for a further 21–24 h to allow ookinete differentiation. Cultures were pelleted for 2 min at 5000 rpm and then incubated with 50 µl of ookinete medium containing Hoechst 33342 DNA dye to a final concentration of 5 µg/ml and a Cy3-conjugated mouse monoclonal antibody 13.1 [16] recognizing the P28 protein on the surface of ookinetes and any undifferentiated macrogametes or zygotes. P28-positive cells were counted with a Zeiss AxioImager M2 microscope (Carl Zeiss, Inc) fitted with an AxioCam ICc1 digital camera. Ookinete conversion was expressed as the percentage of P28 positive parasites that had differentiated into ookinetes [40]. For mosquito transmission experiments 20–50 *Anopheles stephensi* SD500 mosquitoes were allowed to feed for 20 min on anaesthetized infected mice whose asexual parasitaemia had reached ∼5–7% and were carrying comparable numbers of gametocytes as determined on Giemsa stained blood films. Day 14 post feeding approximately 20 mosquitoes were dissected and oocysts on their mid-guts counted. Oocyst formation was examined by Hoechst 33342 staining for 10–15 min and guts were washed and mounted under Vaseline-rimmed cover slips. Images were recorded using a 63× oil immersion objective on a Zeiss AxioImager M2 microscope fitted with an AxioCam ICc1 digital camera. Day 21 post feeding another 20 mosquitoes were dissected and their guts and salivary glands crushed separately in a loosely fitting homogenizer to release sporozoites, which were then quantified using a haemocytometer. Due to day-to-day variations in transmission levels, all data were normalized to a matching number of wild type controls analyzed on the same day.

### Bioinformatic analysis

Using *Arabidopsis thaliana* BSU1 sequence (RefSeq: NP_171844) as a seed, the iterative strategy enacted by the ‘jackhammer’ method of HMMER3 [38] was used to search the predicted proteomes of 46 diverse eukaryotes [60] with the addition of the *Emiliania huxleyi* dataset from JGI (www.jgi.doe.gov). Four iterations were made with an inclusion theshold (e-value) of <10^−25^. 221 sequences matching the final profile at e<10^−120^ were found to form 3 well-defined clusters using the BLAST-clustering approach [61]. Selected sequences were trimmed to 50 aa either side of the phosphatase domain as defined by Pfam domain PF00149 and aligned using MAFFT6.24 [62]. Well-aligned blocks were used to infer a Bayesian phylogeny using the metropolis-coupled Markov chain Monte Carlo (MCMCMC) [63]. Four independent runs of 400,000 generations were performed from random start trees, using the WAG substitution matrix with a gamma-distributed variation in substitution rate approximated to 4 discrete categories (shape parameter estimated from the data). Protein domain architectures were predicted from the models in Pfam25 (A and B) with e-value<10^−3^. Additional orthologues from *Plasmodium* species were identified from PlasmoDB (http://plasmodb.org/).

Phylogenetic trees of the 6 different kelch domains were constructed using kelch phosphatase sequences identified in BLAST searches of eukaryotic genomes. The sequences were aligned using ClustalW2 and optimised using CLC Genomics Workbench (CLC bio, Cambridge, MA). After identifying the 6 kelch-domains the kelch-domain coding sequences were realigned using the same program and neighbour-joining bootstrap trees were generated. The phylogenetic trees were drawn using Fig Tree v1.3.1.

### Purification of schizonts, gametocytes and ookinetes

Purification of gametocytes was achieved using a protocol modified from [64]. Mice were treated by intra-peritoneal injection of 0.2 ml of phenylhydrazine (6 mg/ml) (Sigma) in PBS to encourage reticulocyte formation four days prior to infection with parasites. Day four post infection (p.i.) mice were treated with sulfadiazine (Sigma) at 20 mg/L in their drinking water for two days to eliminate asexual blood stage parasites. On day six p.i. mice were bled by cardiac puncture into heparin and gametocytes separated from uninfected erythrocytes on a 48% NycoDenz gradient (27.6% w/v NycoDenz in 5 mM Tris-HCl, pH 7.20, 3 mM KCl, 0.3 mM EDTA) in coelenterazine loading buffer (CLB), containing PBS, 20 mM HEPES, 20 mM Glucose, 4 mM sodium bicarbonate, 1 mM EGTA, 0.1% w/v bovine serum albumin, pH 7.25. Gametocytes were harvested from the interface and washed twice in RPMI 1640 ready for activation of gamete formation. Blood cells from day 5 p.i. mice were placed in culture for 24 h at 37°C for schizont (with rotation at 100 rpm) and 20°C for ookinete production as described above. Schizonts and ookinetes were purified on a 60% and 63% NycoDenz gradient, respectively and harvested from the interface and washed.

### Quantitative RT-PCR

Schizonts and gametocytes were purified as described above and frozen in Trizol (Sigma) prior to RNA extraction. Asexual blood parasites were extracted as for gametocytes above but on day four p.i. with very low gametocytaemia and no sulfadiazine treatment. RNA was isolated according to manufacturer's instructions. Isolated RNA was treated with DNase I (Promega) and used in reverse transcription reactions (SuperScript III Reverse Transcription kit, Invitrogen) from 500 ng of total RNA.

Gene expression was quantified by SYBR green PCR using Fast mastermix on an ABI 7500 QPCR System (Applied Biosystems). Primers were designed using the PerlPrimer software program [65] to be 18–22 bp in length, with 30–60% GC content, to amplify a region 50–150 bp long and when possible, to bind within 600 bp of the 3′ end of the genes of interest. Primer efficiencies were all between 90–110%, with qRT-PCR resulting in no detectable primer dimers, as determined by dissociation curves. cDNA was diluted 1∶20 with DEPC-treated water before use. Reactions consisted of 3.6 µl of diluted cDNA, 5 µl SYBR green fast mastermix (Applied Biosystems), 0.2 µl each of forward and reverse primer and 1 µl of DEPC water. Cycling conditions were: 95°C for 20 s followed by 40 cycles of 95°C, 3 s, and 60°C, 30 s, followed by dissociation curve. Three biological replicates, with three technical replicates from each biological replicate were performed for each assayed gene. Wild-type gene expression was determined using the comparative cycle threshold method [66], whereas relative quantification in mutant lines was determined using the Pfaffl method [67]. Both methods used *hsp70* (PBANKA_081890) (forward, 5′-GTATTATTAATGAACCCACCGCT-3′; reverse, 5′-GAAACATCAAATGTACCACCTCC-3′) and *arginyl-tRNA synthetase* (PBANKA_143420) (forward, 5′-TTGATTCATGTTGGATTTGGCT-3′; reverse, 5′-ATCCTTCTTTGCCCTTTCAG-3′) as reference genes. *ppkl* primers were: forward, 5′- TTCTAAAGTACCTTCACCAAGAG-3′; reverse, 5′- TAGCAGGTCCTTCTTTACAC-3′. *map2* (PBANKA_093370) primers were: forward, 5′-AATGAAGAACCAGGGCCA-3′; reverse, 5′-ACCATCTAGTAACTACATGGCT-3′. *nek4* (PBANKA_061670) primers were: forward, 5′-CTTCAGATGTATGGGCTATTGG-3′; reverse, 5′- TTCCCTTTGTTGAATGAAATGG-3′. *dozi* (PBANKA_121770) primers were: forward, 5′- GCAAGAATGTCGCAAACAC-3′; reverse, 5′-TCTGAGGAAACTAAACATCGAC-3′.

### Western blotting

Western blot analysis was performed on cell lysates prepared by re-suspending parasite pellets in a 1∶1 ratio of PBS containing Protease inhibitor (Roche) and Laemmli sample buffer, boiling and separating on a 4%–15% SDS-polyacrylamide gel (BioRad). Samples were subsequently transferred to nitrocellulose membranes (Amersham Biosciences) and immunoblotting performed using the Western Breeze Chemiluminescent Anti-Rabbit kit (Invitrogen) and anti-GFP polyclonal antibody (Invitrogen) at a concentration of 1∶1250, according to the manufacturer's instructions.

### Isolation of PPKL-GFP protein and phosphatase assay

Blood aliquots from mice infected with PPKL-GFP and WT parasites were incubated in ookinete medium for 30 min at 20°C and processed as described previously [46]. The resulting parasite pellets were incubated for 30 min at 4°C in lysis buffer (10 mM Tris-HCl, pH 7.5, 150 mM NaCl, 0.5 mM EDTA, 0.5% NP-40) supplemented with protease inhibitors (Roche), then the lysates were centrifuged at 20,000 g for 5 min and the supernatants were subjected to immunoprecipitation using GFP-TRAP beads (ChromoTek) according to manufacturer's instructions. Protein phosphatase activity of the immunoprecipitate was assessed using the SensoLyte MFP Protein Phosphatase Assay Kit (AnaSpec) according to manufacturer's instructions. Briefly, the GFP-TRAP beads were resuspended and diluted in phosphatase assay buffer (100 mM Tris-HCl pH 7.5, 4 mM DTT, 0.2 mM EDTA, 0.5 mM MnCl_2_, 0.4 mg/ml BSA), incubated for 30 min at 37°C in the presence or absence of MFP fluorogenic phosphatase substrate, and centrifuged for 2 min at 2700 g. Supernatants were transferred to a 96-well microplate and the fluorescence generated by the dephosphorylation of MFP was measured using a microplate fluorimeter. The presence of PPKL-GFP in the bead pellets was assessed by Western blot.

### Electron microscopy

Samples cultured in ookinete medium as described above were fixed in 4% glutaraldehyde in 0.1 M phosphate buffer and processed for routine electron microscopy as previously described [68]. Briefly, samples were post fixed in osmium tetroxide, treated *en bloc* with uranyl acetate, dehydrated and embedded in Spurr's epoxy resin. Thin sections were stained with uranyl acetate and lead citrate prior to examination in a JEOL1200EX electron microscope (Jeol UK Ltd).

### Immunofluorescence assay

IFAs were performed on air dried ookinete slides from ookinete cultures produced as previously described [46]. Briefly, infected blood from mice with 7–10% gametocytaemia was incubated for 24 h in ookinete medium at 20°C, then ookinete smear slides were prepared and air dried. For various antibodies different procedures were followed. For α-tubulin, slides were fixed in 4% paraformaldehyde in MTSB buffer [46] and the mouse monoclonal α-tubulin (Sigma) was used as the primary antibody (1∶1000 dilution). For actin staining the procedure of [21] was followed. Briefly, ookinetes were freshly fixed in 4% paraformaldehyde in MTSB with 0.2%Triton, followed by methanol fixation for 5 min and stained with monoclonal Dictyostelium anti-actin antibody (1∶1000 dilution). For GAP45 and MTIP, cells were fixed in 4% paraformaldehyde in MTSB buffer and stained with antibodies against GAP45 (1∶250) and MTIP (1∶250) [69]. For CTRP, air dried slides were fixed for 5 min in 1% formaldehyde and mouse monoclonal anti-CTRP antibody (1∶1000) was used [25]; for anti-SOAP antibodies, slides were fixed in 4% paraformaldehyde and a 1∶100 dilution of antibody was used [26]. For mouse monoclonal antibodies 568 AlexaFluor-labelled anti-mouse (Invitrogen) (1∶1000) was used as a secondary antibody. For GAP45 and MTIP antibodies, AlexaFluor 466 labelled anti-rabbit (Invitrogen) (1∶1000) was used as a secondary antibody.

### PPKL *in vivo* phosphorylation

Blood aliquots from infected mice were incubated overnight, from which schizonts were purified as described previously [46]. Gametocytes were purified and activated for 25 min at 20°C in ookinete medium as described above. Schizonts and activated gametocytes were then washed in phosphate-free Krebs buffer (118 mM NaCl, 4.7 mM KCl, 4.2 mM NaHCO_3_, 1.2 mM MgSO_4_, 11.7 mM glucose, 10 mM HEPES, 1.3 mM CaCl_2_, pH 7.4) and metabolically labelled with 3–5 MBq ^32^P-orthophosphate in phosphate-free Krebs buffer for 30 min at 20°C or 37°C for activated gametocytes and schizonts, respectively. After two washes in phosphate-free Krebs buffer, the labelled parasites were lysed for 30 min at 4°C in lysis buffer (10 mM Tris-HCl pH 7.5, 150 mM NaCl, 0.5 mM EDTA, 0.5% NP-40) supplemented with protease and phosphatase inhibitors (Roche), the resulting lysate was centrifuged at 20,000 g for 5 min and the supernatant collected. PPKL-GFP protein was isolated using GFP-TRAP beads (ChromoTek), the immunoprecipitated proteins were then resuspended in Laemmli sample buffer and separated by SDS-PAGE. ^32^P-labelled proteins were visualized using a phosphorimager (Molecular Dynamics) and GFP-tagged proteins analysed by Western blot as described above. The relative PPKL-GFP phosphorylation levels in activated gametocytes with respect to schizonts were obtained by taking the normalized ratio between the intensity of the phosphorylation signal from the phosphoimager and the intensity of the GFP immunoreactive signal from the corresponding Western Blot by using the ImageJ software (National Institute of Health).

### Metabolic labelling for global phosphorylation profile

Gametocytes from wild type, *ppkl^−^* and *nek4^−^* mutant parasites were purified as described above from the blood of infected mice. Purified gametocytes were placed for 60 min, 5.5 h and 23.5 h in ookinete medium at 20°C to activate both male and female gametocytes to form gametes. For metabolic labelling, the parasites were washed once with 1 ml of phosphate-free Kreb's buffer and resuspended in 500 µl of the same buffer. 20–25 µl ^32^P-orthophosphate (7–9.25 MBq) was added to the suspension and parasites incubated at 20°C for 30 min. The labelled parasites were then lysed in lysis buffer (50 mM Tris, 0.5 mM EDTA, 5% β-glycerolphosphate, pH 7.6, supplemented with protease/phosphatase inhibitors (Roche) and 1% NP-40). Following incubation on ice for 10 min, the samples were centrifuged for 3 min at 20000 g and the supernatants were collected for further fractionation. Fractionation was carried out on an AKTA chromatographer (Amersham Pharmacia Biotec) using Resource Q (Amersham Pharmacia Biotec) anion-exchange column (matrix volume 1 ml). The proteins were eluted using a linear gradient of 0–1.0 M NaCl in running buffer (10 mM Tris, 5 mM EDTA and 20 mM β-glycerolphosphate, pH 7.4). Fractions (1 ml) were collected and analysed further by resolution on SDS-PAGE gels. ^32^P-labelled proteins were visualised by autoradiography.

### Injections of ookinetes into the mosquito haemocoel

Ookinetes were cultured using standard methods and harvested by pelleting. *A. stephensi* mosquitoes were anaesthetised with CO_2_ and approximately 500 ookinetes in 69 µl of complete ookinete medium were injected into the thorax using a microinjector (Drummond, Nanoject II). Bitebacks and dissections for quantification of salivary gland sporozoites were performed 20 days after injection.

### Statistical analyses

All statistical analyses were performed using GraphPad Prism (GraphPad Software). For ookinete motility analysis, non-parametric t-tests were used. For relative quantification of qRT-PCR reactions, a pair-wise fixed reallocation randomisation test [67] was used.

### Accession numbers

Sequences are derived from Uniprot (http://www.uniprot.org/), *P. berghei* (Q4Z2M2), *P. yoelii* (Q7RA97), *P. chabaudi* (Q4XN17), *P. falciparum* (Q8IKH5), *P. knowlesi* (B3L999), *P. vivax* (A5K396), *T. gondii* (B6KKQ9), *T. parva* (Q4N2C8), *C. muris* (B6A994), *T. thermophila* (Q22BC4), *C. reinhardtii* (A8HNE3), *A. thaliana* (Q8L7U5), *P. marinus* (C5K554), *B. bovis* T2Bo (A7AT59), *C. variabilis* (E1ZIE2), *V. carteri f. nagariensis* (D8UHM6), *S. moellendorffii* (D8RUC3), *P. tetraurelia* strain d42 (A0CLB3), *M. pusilla* CCMP1545 (C1MQB3), *O. lucimarinus* CCE9901 (A4S3K6), *N. caninum* (F0VC41), *I. multifiliis* (G0QV54).

## Supporting Information

Figure S1 **Bioinformatic analysis of individual PPKL kelch domains in** ***Plasmodium.*** The *P. berghei* Kelch repeat protein phosphatase contains five full (A–E, kelch 1–5) and one truncated (F, kelch 6) kelch repeats. The trees were constructed using Kelch phosphatase sequences identified in BLAST searches of eukaryotic genomes. The sequences were aligned using ClustalW2 and optimised using CLC Genomics Workbench. After identifying the 6 kelch-domains the kelch-domain coding sequences were realigned using the same program and neighbour-joining bootstrap trees were generated. The phylogenetic trees were drawn using Fig Tree v1.3.1.
(TIF)

Figure S2 **Sequence alignment of the protein phosphatase domains of kelch phosphatases from** ***Alveolata*** **and** ***Viridiplantae*** **.** The invariant residues conserved in protein phosphatases are highlighted in grey and metal ion binding sites are marked with solid circle. The amino acids with role in inhibitor binding are highlighted: Inhibitor-2 binding amino acids (1–7. blue squares); microcystin binding amino acids (1–4. orange squares); okadaic acid and microcystin binding amino acids (1–5. green squares); Inhibitor-2, microcystin, okadaic acid binding amino acid (1–2. blue boxes). Conserved insertions identified in Alveolates are marked with black bars (I–IV) above the sequences. *A. thaliana* and *P. berghei* type 1 protein phosphatases (PP1) were included in the alignment as markers for the PP1 catalytic subunit.
(TIF)

Figure S3 ***gfp*** **tagging of the endogenous** ***ppkl*** **locus.** A. Schematic representation of the gene targeting strategy used for gene tagging the endogenous locus with *gfp* via single homologous recombination. Primers 1+2 used for diagnostic PCR are indicated, as well as the *EcoR*I site used for Southern blotting. Probe location used for detection by Southern blotting is indicated. B. Diagnostic PCR confirming successful integration of the tagging sequence. C. Southern blot analysis of *EcoR*I digested *ppkl* genomic DNA using the 3′ UTR of the targeting construct as a probe. Band sizes for PPKL-GFP (*tag*) and wild-type (*wt*) are indicated. D. Pulse-field gel electrophoresis blot hybridized with *Pb* 3′UTR which detects the endogenous chromosome 7 locus and *hdhfr* of the C-fusion *gfp* sequence integrated on chromosome 13–14. E. Western blot analysis using an anti-GFP antibody against control wild-type-GFP (wt) and transgenic (tag) ookinetes (ook) non-activated gametocytes (NAG) and activated gametocytes (AG) showing bands of expected sizes of 29 kDa for wild-type-GFP and 128 kDa for PPKL-GFP.
(TIF)

Figure S4 **Deletion of the** ***ppkl*** **gene.** A. Schematic representation of the gene targeting strategy used for gene disruption via double homologous recombination. Primers 1–4 used for diagnostic PCR are indicated, as well as the *EcoR*I digestion site used for Southern blotting. Probe location used for detection by Southern blotting is indicated. B. Diagnostic PCR confirming successful integration of the disruption sequence of *ppkl* in mutant clone 3 (*cl3*), clone 9 (*cl9*) and a third clone in the *P. berghei* ANKA 2.34 line constitutively expressing GFP (clone 6 – *cl6 GFP*). Primers 1+2 were used to verify successful integration at the correct locus. Primers 3+4 were used to confirm loss of the endogenous gene. C. Southern blot analysis of *EcoR*I digested clone 3 and 9 genomic DNA using the 3′ UTR of the targeting construct as a probe. Band sizes for *ppkl^−^* clone 3 (*cl3*), clone 9 (*cl9*) and wild-type (*wt*) are indicated. D. Pulse-field gel electrophoresis blot hybridized with *Pb* 3′UTR which detects the endogenous chromosome 7 locus and disrupted locus on chromosome 13 in both clones. E. Bar graph showing relative expression of endogenous *ppkl* in *ppkl^−^* mutants using qRT-PCR compared to wild-type. Error bars = ±SEM, *n = *3 from three separate experiments in both clone 3 and clone 9.
(TIF)

Figure S5 **Montage of wild-type and** ***ppkl^−^*** **parasites.** A. Montage of longitudinal sections through the apex of six different wild-type ookinetes illustrating the similarity in appearance of the apical structures. Bar = 100 nm. B. Montage of longitudinal sections of the anterior of *ppkl^−^* mutants showing the variability of appearances ranging from relatively normal to severe collapse. In all cases the electron dense collar appeared to be reduced in size. Bar = 100 nm. C. Correlation of ultrastructural (TEM) and immunocytochemical (Hoechst and P28 staining) appearances of a variety of different *ppkl^−^* morphologies after *in vitro* culture in ookinete medium for 24 h. Bar = 1 µm.
(TIF)

Video S1 **Wild-type ookinete motility.**
(MOV)

Video S2 ***ppkl^−^*** **retort motility.**
(MOV)
