# Supplementary figures and images for: A Unique Protein Phosphatase with Kelch-Like Domains (PPKL) in Plasmodium Modulates Ookinete Differentiation, Motility and Invasion
Source: PLoS Pathog. 2012 Sep 20;8(9):e1002948. doi: 10.1371/journal.ppat.1002948 (PMC3447748; doi:10.1371/journal.ppat.1002948)

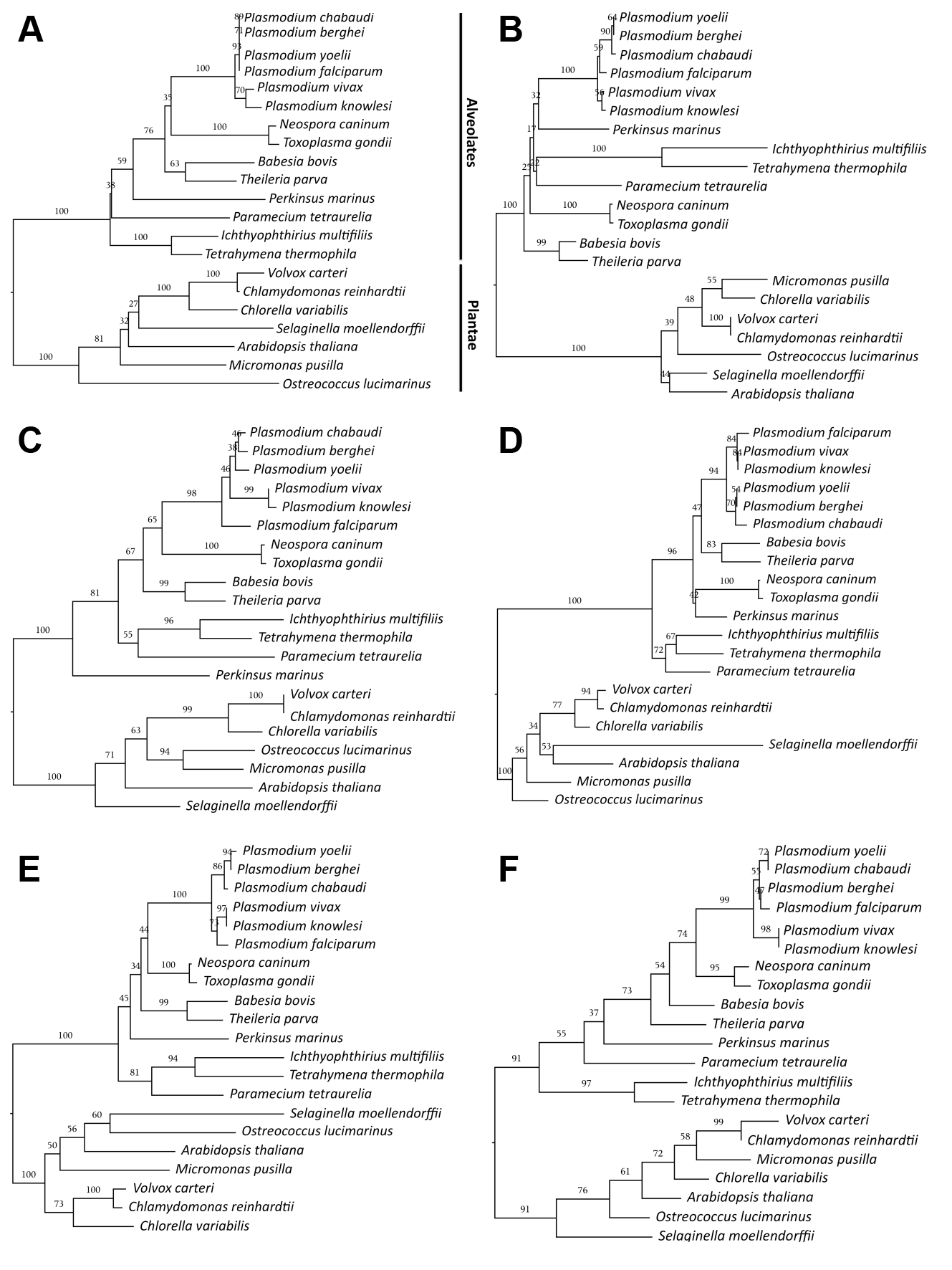

Supplement: Figure S1 — Bioinformatic analysis of individual PPKL kelch domains in Plasmodium. The P. berghei Kelch repeat protein phosphatase contains five full (A–E, kelch 1–5) and one truncated (F, kelch 6) kelch repeats. The trees were constructed using Kelch phosphatase sequences identified in BLAST searches of eukaryotic genomes. The sequences were aligned using ClustalW2 and optimised using CLC Genomics Workbench. After identifying the 6 kelch-domains the kelch-domain coding sequences were realigned using the same program and neighbour-joining bootstrap trees were generated. The phylogenetic trees were drawn using Fig Tree v1.3.1. (TIF) [file ppat.1002948.s001.tif]

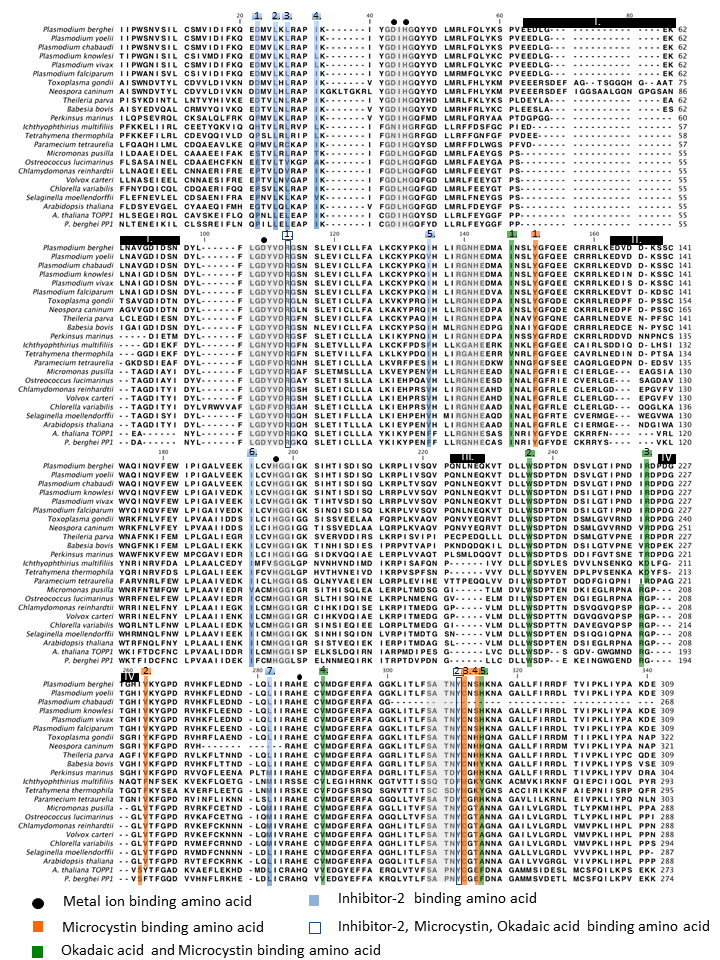

Supplement: Figure S2 — Sequence alignment of the protein phosphatase domains of kelch phosphatases from Alveolata and Viridiplantae . The invariant residues conserved in protein phosphatases are highlighted in grey and metal ion binding sites are marked with solid circle. The amino acids with role in inhibitor binding are highlighted: Inhibitor-2 binding amino acids (1–7. blue squares); microcystin binding amino acids (1–4. orange squares); okadaic acid and microcystin binding amino acids (1–5. green squares); Inhibitor-2, microcystin, okadaic acid binding amino acid (1–2. blue boxes). Conserved insertions identified in Alveolates are marked with black bars (I–IV) above the sequences. A. thaliana and P. berghei type 1 protein phosphatases (PP1) were included in the alignment as markers for the PP1 catalytic subunit. (TIF) [file ppat.1002948.s002.tif]

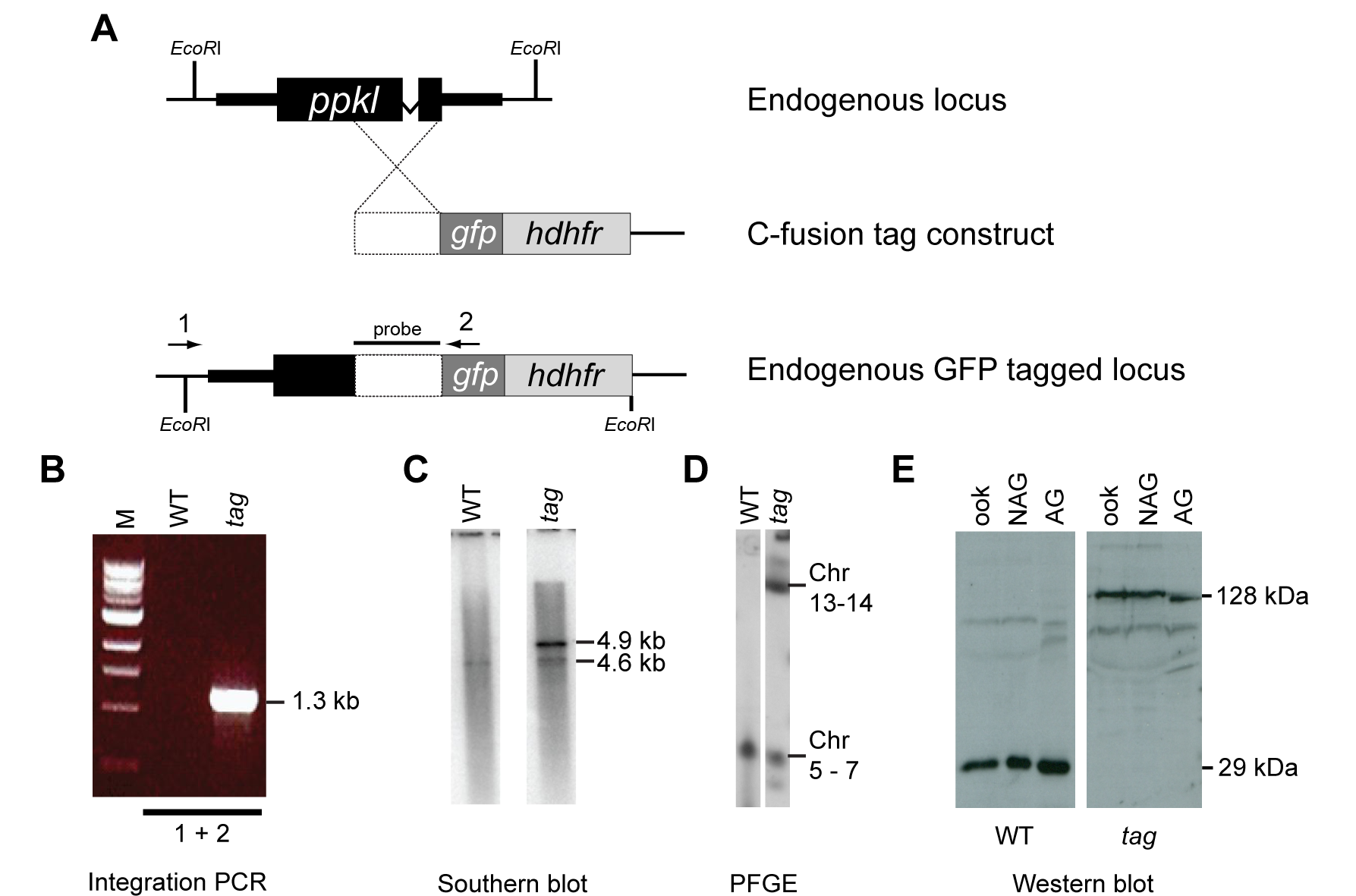

Supplement: Figure S3 — gfp tagging of the endogenous ppkl locus. A. Schematic representation of the gene targeting strategy used for gene tagging the endogenous locus with gfp via single homologous recombination. Primers 1+2 used for diagnostic PCR are indicated, as well as the EcoRI site used for Southern blotting. Probe location used for detection by Southern blotting is indicated. B. Diagnostic PCR confirming successful integration of the tagging sequence. C. Southern blot analysis of EcoRI digested ppkl genomic DNA using the 3′ UTR of the targeting construct as a probe. Band sizes for PPKL-GFP (tag) and wild-type (wt) are indicated. D. Pulse-field gel electrophoresis blot hybridized with Pb 3′UTR which detects the endogenous chromosome 7 locus and hdhfr of the C-fusion gfp sequence integrated on chromosome 13–14. E. Western blot analysis using an anti-GFP antibody against control wild-type-GFP (wt) and transgenic (tag) ookinetes (ook) non-activated gametocytes (NAG) and activated gametocytes (AG) showing bands of expected sizes of 29 kDa for wild-type-GFP and 128 kDa for PPKL-GFP. (TIF) [file ppat.1002948.s003.tif]

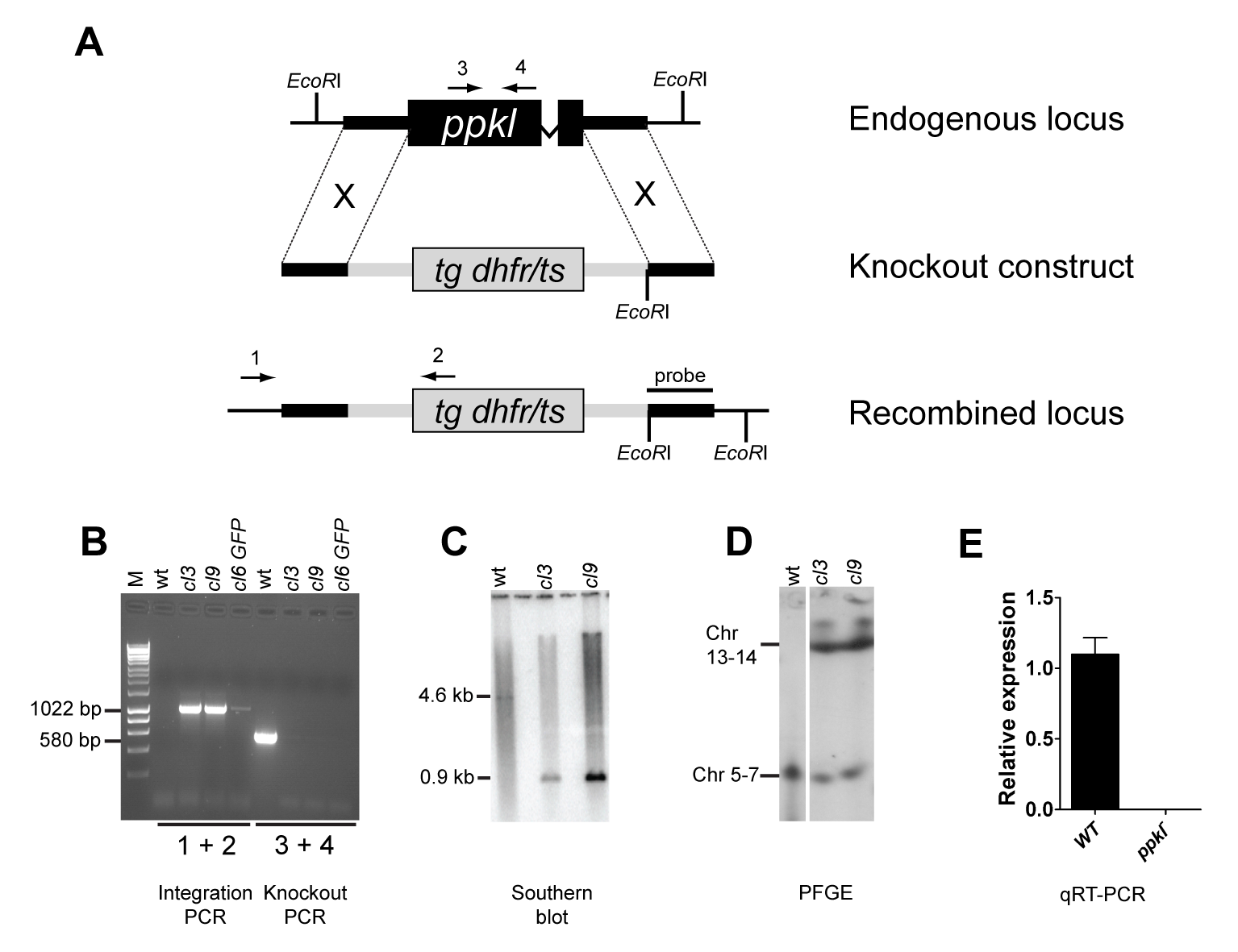

Supplement: Figure S4 — Deletion of the ppkl gene. A. Schematic representation of the gene targeting strategy used for gene disruption via double homologous recombination. Primers 1–4 used for diagnostic PCR are indicated, as well as the EcoRI digestion site used for Southern blotting. Probe location used for detection by Southern blotting is indicated. B. Diagnostic PCR confirming successful integration of the disruption sequence of ppkl in mutant clone 3 (cl3), clone 9 (cl9) and a third clone in the P. berghei ANKA 2.34 line constitutively expressing GFP (clone 6 – cl6 GFP). Primers 1+2 were used to verify successful integration at the correct locus. Primers 3+4 were used to confirm loss of the endogenous gene. C. Southern blot analysis of EcoRI digested clone 3 and 9 genomic DNA using the 3′ UTR of the targeting construct as a probe. Band sizes for ppkl− clone 3 (cl3), clone 9 (cl9) and wild-type (wt) are indicated. D. Pulse-field gel electrophoresis blot hybridized with Pb 3′UTR which detects the endogenous chromosome 7 locus and disrupted locus on chromosome 13 in both clones. E. Bar graph showing relative expression of endogenous ppkl in ppkl− mutants using qRT-PCR compared to wild-type. Error bars = ±SEM, n = 3 from three separate experiments in both clone 3 and clone 9. (TIF) [file ppat.1002948.s004.tif]

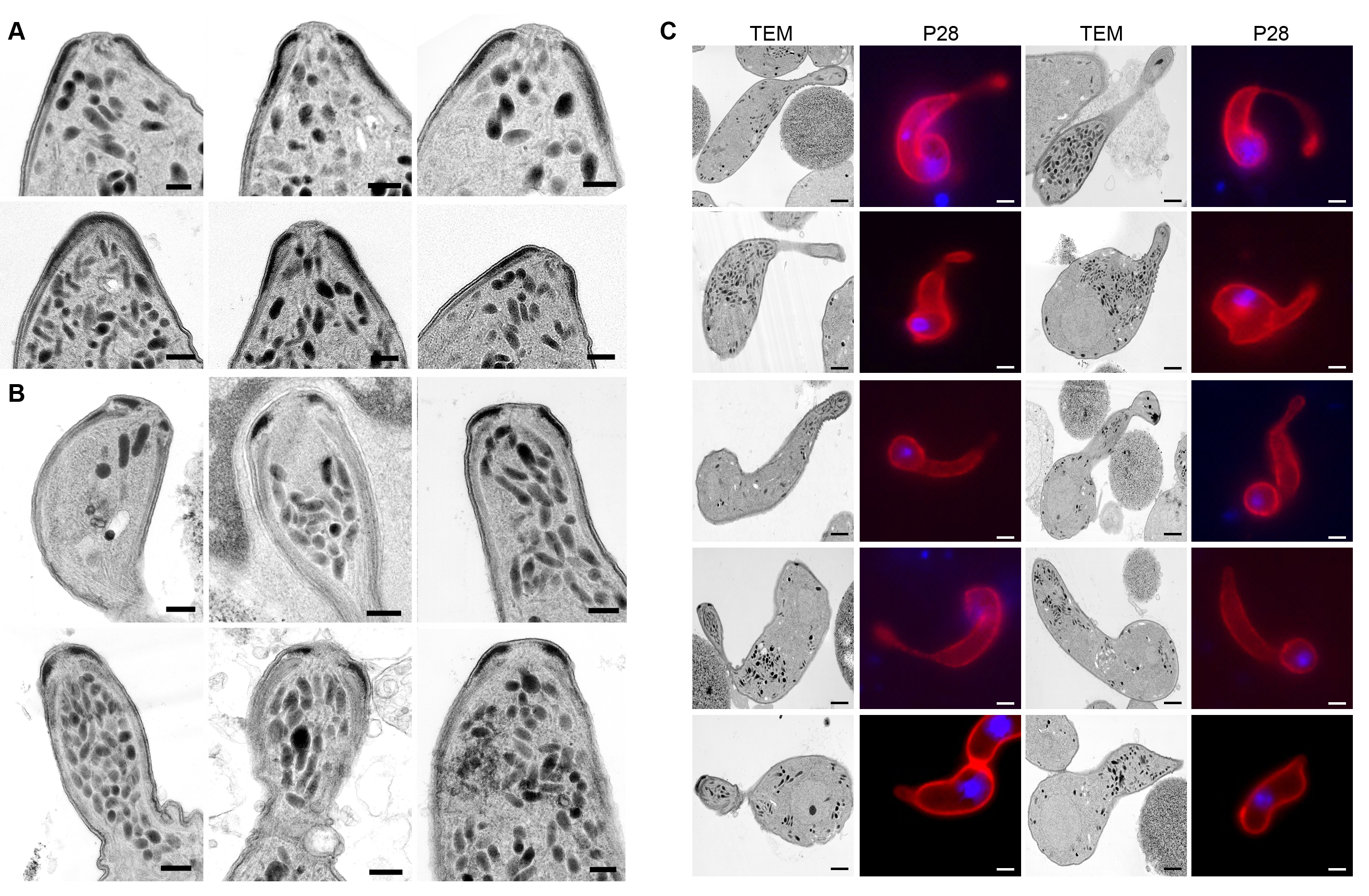

Supplement: Figure S5 — Montage of wild-type and ppkl− parasites. A. Montage of longitudinal sections through the apex of six different wild-type ookinetes illustrating the similarity in appearance of the apical structures. Bar = 100 nm. B. Montage of longitudinal sections of the anterior of ppkl− mutants showing the variability of appearances ranging from relatively normal to severe collapse. In all cases the electron dense collar appeared to be reduced in size. Bar = 100 nm. C. Correlation of ultrastructural (TEM) and immunocytochemical (Hoechst and P28 staining) appearances of a variety of different ppkl− morphologies after in vitro culture in ookinete medium for 24 h. Bar = 1 µm. (TIF) [file ppat.1002948.s005.tif]
